# How Speededness of a Reasoning Test and the Complexity of Mental Speed Tasks Influence the Relation between Mental Speed and Reasoning Ability

**DOI:** 10.3390/jintelligence11050089

**Published:** 2023-05-08

**Authors:** Natalie Borter, Katja Schlegel, Stefan J. Troche

**Affiliations:** Institute of Psychology, University of Bern, Fabrikstrasse 8, CH-3012 Bern, Switzerland; katja.schlegel@unibe.ch (K.S.); stefan.troche@unibe.ch (S.J.T.)

**Keywords:** reasoning, mental speed, task complexity, speededness

## Abstract

Although previous research has consistently reported a positive association between mental speed and reasoning ability, it remains unclear whether the magnitude of this association depends on whether the reasoning test is administered with or without a time limit. In addition, it is unknown how mental speed task complexity affects the mental speed–reasoning association when the effects of time limitations in the reasoning test (labeled “speededness”) are controlled for. The present study examined these questions in a sample of 200 participants who completed the time-limited Culture Fair Test (CFT) and a Hick task with three levels of complexity to measure mental speed. Results showed that the latent correlation between mental speed and reasoning was slightly lower when the effect of speededness in reasoning was statistically controlled for. However, for both controlled and uncontrolled reasoning, the correlation with mental speed was of medium size and statistically significant. When reasoning was controlled for the effects of speededness, only complexity-related mental speed aspects were correlated with reasoning, whereas basic mental speed aspects were correlated with the speededness factor and unrelated to reasoning. These findings demonstrate that time limitations in reasoning tests and complexity in mental speed tasks affect the magnitude of the mental speed–reasoning association.

## 1. How Speededness of a Reasoning Test and the Complexity of Mental Speed Tasks Influence the Relation between Mental Speed and Reasoning Ability

Individuals with higher psychometric intelligence have been consistently reported to process information faster than individuals with lower intelligence scores (Danthiir et al. 2005). Although current models of intelligence differentiate between “reaction and decision speed” on the one hand, and “processing speed” on the other one (McGrew 2009), there is some evidence for the notion that both aspects of speed tap the same construct and their differentiation is primarily caused by the assessment through reaction times and number of items processed within a given time (Schmitz and Wilhelm 2019).

Commonly, reaction/decision speed is measured by elementary cognitive tasks (ECTs), which are computer-based and involve a limited number of specific mental operations, placing low demands on motor preparation and execution (Danthiir et al. 2005; Jensen 1998). Reaction times (RTs) in these ECTs are used as indicators for mental speed and show a quite consistent negative association with psychometric intelligence (Sheppard and Vernon 2008; Doebler and Scheffler 2016). This relation seems to increase with increasing complexity of a given ECT (Danthiir et al. 2005; Pahud et al. 2018). However, the strength of the association and its role in explaining individual differences in intelligence remain a source of continuous debate (Anderson and Nelson 2005; Jensen 2006; Schmitz and Wilhelm 2016; Stankov and Roberts 1997).

Many studies in this field use time-limited tests to measure intelligence or, more specifically, reasoning ability as a well-established proxy of general intelligence (Johnson et al. 2008; Kan et al. 2011). Such a time limitation is common for psychometric intelligence tests as it increases test efficiency and facilitates the testing of groups of participants in research. Surprisingly, however, only a very limited number of studies (described in detail below) investigated whether the time limitation and the resulting speededness of the test influence the relation between mental speed and intelligence (Vernon and Kantor 1986; Wilhelm and Schulze 2002). In this context, speededness refers to the degree that test performance is affected by a limited testing time, i.e., when (at least some) participants do not have enough time to attempt all items or guess, especially on the last items of a test (Estrada et al. 2017; Lu and Sireci 2007). Hence, the relationship between psychometric intelligence and RT measures in ECTs could be overestimated when speed of information processing influences both the RT measures and, as a kind of method effect, the measure of intelligence (Wilhelm and Schulze 2002).

In one of the few existing studies examining this question, Vernon and Kantor (1986) asked participants to complete a set of computer-based ECTs with varying complexity. Additionally, one half of the sample completed a battery of time-limited intelligence tests, while the other half completed a battery of time-unlimited intelligence tests. The correlation between a mental speed factor extracted from the ECTs and intelligence was slightly (but not significantly) higher for time-unlimited than for time-limited intelligence tests (see also Vernon et al. 1985). Thus, in this study, time limitations of the intelligence tests had no influence on the correlational relationship between mental speed and intelligence. It should be noted, however, that time-unlimited intelligence tests correlated most strongly with RT in the most complex ECTs, defined as those ECTs with the highest mean RTs. In contrast, the time-limited intelligence tests correlated most strongly with RTs in those ECTs that had the highest loadings on a general mental speed factor and presumably captured the largest variety of basic information-processing operations (Vernon and Kantor 1986).

In another study, a sample of 367 participants completed a battery of time-limited and a battery of time-unlimited reasoning tasks along with a battery of paper–pencil-based clerical speed tests (Wilhelm and Schulze 2002). Latent variables representing time-limited and time-unlimited reasoning ability as well as mental speed were extracted from the respective tests. Both time-limited and time-unlimited reasoning ability were related to mental speed. In contrast to Vernon and Kantor (1986), however, the relation to mental speed was stronger for time-limited compared to time-unlimited reasoning ability. Wilhelm and Schulze (2002) concluded that time-limited reasoning scores are a compound of reasoning ability and mental speed, which leads to an artificial overestimation of the true relation between reasoning ability and mental speed.

The above-mentioned studies were very demanding in terms of data collection, with Vernon and Kantor (1986) requiring two large groups to complete either time-unlimited *or* time-limited tests and with Wilhelm and Schulze (2002) administering a very large battery of time-limited and time-unlimited tests to one group. A more parsimonious approach to isolate test speededness recently introduced by Schweizer and colleagues (Ren et al. 2013, 2018; Zeller et al. 2018) is based on confirmatory factor analysis (CFA). This approach uses bifactor modeling to separate a latent variable representing the effect of speededness from a latent variable representing reasoning as a measure of intelligence (see method section for a detailed statistical explanation). The advantage of this approach is that the intelligence assessment does not need to be presented under both time-limited and time-unlimited conditions. Since the effect of speededness is statistically controlled for in such a CFA model, reasoning ability can be estimated in a way that is purified from the effect of speededness on test performance (Borter et al. 2020).

In one of the initial studies using this approach, Ren et al. (2018) used Raven’s Advanced Progressive Matrices as a measure of reasoning ability with a time limitation and separated reasoning ability from the effect of speededness. An additional paper–pencil-based clerical speed measure, where specific target items presented among distractors had to be identified as fast as possible, correlated with the effect of speededness but not with reasoning ability. Thus, similar to Wilhelm and Schulze’s (2002) results, this finding suggests that the time limitation of reasoning tests leads to an overestimation of the relation between mental speed and reasoning ability or intelligence more generally.

However, it is still not clear whether clerical speed measures in paper–pencil tests (e.g., the number of correctly identified targets among distractors on a paper sheet in a given time) functionally tap the same ability as RT measures in ECTs (Carroll 1993; Schmitz and Wilhelm 2019). Therefore, the first research question of the present study is to assess how the effect of speededness affects the relationship between reasoning ability and mental speed represented by RTs in the Hick task as a well-established ECT. For this purpose, the relationship between reasoning ability in Cattell’s time-limited Culture Fair Test (CFT) and mental speed is computed twice. Initially, the effect of speededness on CFT performance is not controlled for; however, subsequently, it is statistically controlled for by means of a bifactor CFA model. Based on the findings by Wilhelm and Schulze (2002) and Ren et al. (2018), we expect a weaker association between reasoning and mental speed when speededness is controlled for.

The second research question of the present study is to assess how increasing the complexity of the ECT (operationalized through an increasing number of choices in the Hick task) affects the relationship between mental speed and reasoning ability (measured with the CFT) when reasoning ability is controlled for the effect of speededness. To this end, we use a second bifactor CFA model to statistically separate basic and complexity-related mental speed aspects from the RTs in the Hick task. This model is then combined with the bifactor model separating reasoning ability and speededness as explained above. Vernon and Kantor (1986) reported that RTs from more complex ECTs were more closely related to time-unlimited intelligence scores than RTs from less complex ECTs. Proceeding from this finding, we expect that complexity-related aspects of mental speed are more highly correlated with reasoning (controlled for the effect of speededness) than basic mental speed.

Taken together, the present study seeks to clarify the role of mental speed in psychometric intelligence, which has been a longstanding issue in the field. In addition, the study is relevant for applied settings in which intelligence is typically assessed, as it may affect the interpretation of test results.

## 2. Method

### 2.1. Participants

Participants were 67 men and 133 women ranging in age from 17 to 30 years (*M* = 22.6, *SD* = 2.5 years), who were also subjects in the study by Borter et al. (2018) on another research question. A total of 149 participants were university students and 51 participants did not have secondary education. All participants reported normal or corrected-to-normal vision. The study was approved by the local ethics committee. Prior to testing, participants gave informed consent.

### 2.2. Measure of Intelligence

The German adaptation of Cattell’s Culture Fair Test 20-R (CFT; Weiß 2006) was used with a reliability of .92 (Weiß 2006). The CFT is a (not culturally independent) measure of general reasoning ability and fluid intelligence (Carroll 1993; Cattell 1963; Johnson et al. 2008) and consists of four subtests presented in a multiple-choice format with five response options for each item.

In the subtest Series, participants chose the option best completing a series of three figures. In the Classifications subtest, participants were asked to identify the deviant in five figures. In the Matrices subtest, the appropriate figure to complete a matrix had to be found. In the Topologies subtest, participants identified the one figure in which a given number of dot(s) could be placed with the same topological relationship to the other parts as given in a reference configuration.

The first three subtests consisted of 15 items and Topologies consisted of 11 items. In accordance with the standard instructions provided in the test manual, the time limit was set to four minutes for Series and Classifications, and to three minutes for Matrices and Topologies; all items and subtests were presented in the same order for each participant. Responses on all items were scored as 1 (correct option chosen) or 0 (incorrect or no option chosen). In addition, IQ scores were calculated based on the norms in the CFT (Weiß 2006) in order to describe the intelligence level of the sample.

### 2.3. Measurement of Mental Speed

The Hick task was adapted from Neubauer et al. (1992) and programmed in E-Prime Professional 2.0 (Schneider et al. 2002). Stimuli were rectangles with a height of 1.5 cm and a width of 1.9 cm as well as a plus sign with a height and a width of 0.8 cm. They were presented in white against a black background on a 19-inch monitor screen (Belinea, Model 101902) with a refresh rate of 60 Hz. The distance between the participants’ heads and the monitor screen was about 60 cm. The response pad was a Cedrus RB Box, allowing us to record RTs with a resolution of ±1 ms.

The task consisted of three conditions (0-bit, 1-bit, and 2-bit conditions), presented in three blocks of increasing task demands. Each condition consisted of 32 trials preceded by 8 practice trials. In each trial of the 0-bit condition, a black screen was presented for one second. Then, a rectangle appeared in the center of the monitor screen and after a foreperiod of 1000, 1333, 1666, or 2000 ms, a plus sign was presented in the center of the rectangle. Participants were asked to press a designated key on the response pad as soon as the plus sign appeared. The plus sign remained on the screen up until the key press and then disappeared.

The trials of the 1-bit and 2-bit conditions were similar to the 0-bit condition, but two rectangles were presented in the 1-bit condition, and four rectangles in the 2-bit condition. In each trial, the plus sign appeared in one of the rectangles according to a random sequence, which was identical for all participants. Participants were instructed to press a designated key corresponding to the position of the plus sign as soon as the sign appeared. In all conditions, key presses before stimulus appearance and incorrect responses were registered as errors and followed by a 200 ms feedback tone.

The instructions were presented on the screen and encouraged participants to respond as fast as possible but not to sacrifice accuracy. As the dependent variable, the mean latency of correct responses was recorded for each task condition. To minimize the probability of outliers, for each participant, the very first item and the item with the longest response latency were excluded from analysis. Thereafter, a visual outlier control was performed, identifying response latencies under 100 ms and over 1100 ms as outliers and excluding them from further analysis. Less than 0.21% of responses per condition were outliers. In addition, to avoid outliers in mean response latencies, after a visual inspection, mean response latencies were winsorized to 450 ms (*n* = 4) in the 0-bit condition, to 500 ms in the 1-bit condition (*n* = 2), and to 600 ms in the 2-bit condition (*n* = 3).

### 2.4. Time Course of the Study

Participants first completed the CFT, then completed the Hick task and two additional experimental tasks unrelated to the present study. The order of the three experimental tasks was balanced across participants. Each participant was tested individually.

### 2.5. Statistical Analyses

Analyses were conducted using the lavaan package (Rosseel 2012) in the statistical Software R (version 3.2.0). Unless otherwise stated, the reported parameters were standardized.

In a first step, we computed the correlational relationship between reasoning ability (as a proxy of psychometric intelligence) and mental speed without controlling for the effect of speededness. From the items of each CFT subtest, a latent variable representing reasoning ability was extracted using (essentially tau-equivalent) one-factor models. These first-order latent variables were combined to create a second-order latent variable of reasoning ability. For this second-order latent variable, which was uncontrolled for the effect of speededness, we computed the correlation with mental speed as a latent variable extracted from RTs in the three conditions of the Hick task.

In a second step, it was probed whether the effect of speededness could be identified and represented by a latent variable in the four CFT subtests by means of bifactor models. From the four latent variables representing (first-order) reasoning ability as well as the four latent variables representing the (first-order) effect of speededness, second-order latent variables of reasoning ability and the effect of speededness were derived. Again, the correlations between mental speed as a latent variable extracted from RTs in the three Hick task conditions and (second-order) reasoning ability as well as the (second-order) effect of speededness were computed. The correlations between mental speed and reasoning ability were then compared to the respective correlations from the first step, where the effect of speededness was not controlled for.

The CFA modeling of reasoning ability and the effect of speededness need some further explanation. Variances and probability-based covariances between the binary items (1 = correct, 0 = incorrect or no response) served as input for the analyses of the CFT subtests (Schweizer 2013). This input has been shown to outperform tetrachoric correlations in a previous simulation study, especially for sample sizes smaller than *n* = 1000 (Schweizer et al. 2015). For the (essentially tau-equivalent) one-factor models in the first step, one latent variable representing reasoning ability was extracted from the items of each CFT subtest with the factor loadings being fixed to the same value (“1”) assuming that all items measured reasoning in the same way.

For the bifactor models in the second step, the effect of speededness was added as a latent variable to the tau-equivalent model using the procedure suggested by Schweizer et al. (2019b). More specifically, these authors proceeded from the assumption of normally distributed individual differences in the speededness of test taking, which should lead to an approximatively cumulative increase in omissions. When at least one omission was recorded for an item, it was assumed to be affected by speededness. In order to depict the assumed cumulative increase, the factor loadings of the latent variable reflecting the effect of speededness were fixed to follow the course of the increasing logistic function from the first item in a subtest affected by speededness (i.e., the first item for which at least one response was omitted and not followed by responses on subsequent items) to the last item (Schweizer et al. 2019a). Thus, factor loadings were fixed according to the following formula:

(1) $$\lambda \left(i\right)=\frac{e^{i-j}}{{1+e}^{i-j}}$$

where *i* is the position of an item within all items, which were not reached by at least one participant, and *j* is a constant that may be selected to optimize model fit (Schweizer et al. 2019a). As in previous work with the CFT 20-R (Borter et al. 2020), *j* was set to 1.5 for all subscales.

To bridge the gap between binary manifest and continuous latent variables as well as their distributional differences, the factor loading of each item was weighted by the standard deviation (*SD*) of the respective item (Schweizer 2012):

(2) $$SD=\sqrt{p\cdot \left(1-p\right)}$$

The two latent variables in the bifactor models were assumed to be independent from each other and, thus, their correlation was set to zero.

To investigate the second research question of how ECT complexity affects the relation between mental speed and reasoning ability as well as the effect of speededness, the effect of increasing task complexity on RTs had to be modeled. For this purpose, a fixed-links modeling approach was used to extract two latent variables from RTs in the Hick task (Pahud et al. 2018; Troche et al. 2018). To represent individual differences in RTs increasing as a function of task complexity, the first latent variable had linearly increasing factor loadings of 1, 2, and 4. The second latent variable captured individual differences in RTs, which did not vary between the Hick task conditions. Therefore, the factor loadings of this latent variable were fixed to “1”. The correlation between the two latent variables was set to “0”. After having separated basic and complexity-related mental speed for the Hick task, correlations between these latent variables and reasoning ability as well as the effect of speededness were computed.

The confirmatory factor models were evaluated as good (or acceptable) when the root mean square error of approximation (RMSEA) was below 0.08 (Schermelleh-Engel et al. 2003) and the Standardized Root Mean Square Residual (SRMR) below 0.10 (Schermelleh-Engel et al. 2003). The comparative fit index (CFI) was not used for the analyses on the CFT, as it is non-informative when the investigated model is compared to a too-well-fitting baseline model (Kenny 2015). Kenny (2015) suggested a RMSEA below .158 as being indicative of a too-well-fitting baseline model, which was the case with all four baseline models of the CFT (*Series*: RMSEA = .083; *Classification*: RMSEA = .074; *Matrices*: RMSEA = .094; *Topologies*: RMSEA = .110). To compare models, the Akaike Information Criterion (AIC) was used. A lower AIC indicates a better model with respect to both model fit and parsimony.

## 3. Results

Descriptive statistics of all measures are presented in Table 1. All variables can be assumed to be approximately normally distributed as skewness was below 2 and kurtosis below 7 (Hair et al. 2010; Byrne 2010). The sum of correct responses in the four CFT scores was transferred to age-stratified IQ scores in accordance with the manual. With *M* = 110, the mean IQ of the present sample was higher than the population mean of 100, but the standard deviation (*SD* = 14) was close to the standard deviation of 15 in the population.

The manipulation of task complexity in the Hick task was successful as indicated by the systematic increase in the mean RT across the three Hick task conditions, *F*(1.55,309) = 953.11, *p* < .001, η^2^ = .83. The degrees of freedom were adjusted due to violations of sphericity (Greenhouse and Geisser 1959). Bonferroni-corrected *t*-tests revealed that all three task conditions differed significantly from each other, all *t*s(199) > 13, *p* < .001.

### 3.1. The Relationship between Mental Speed and Reasoning Ability When Controlled and When Not Controlled for the Effect of Speededness

**Figure 1:**
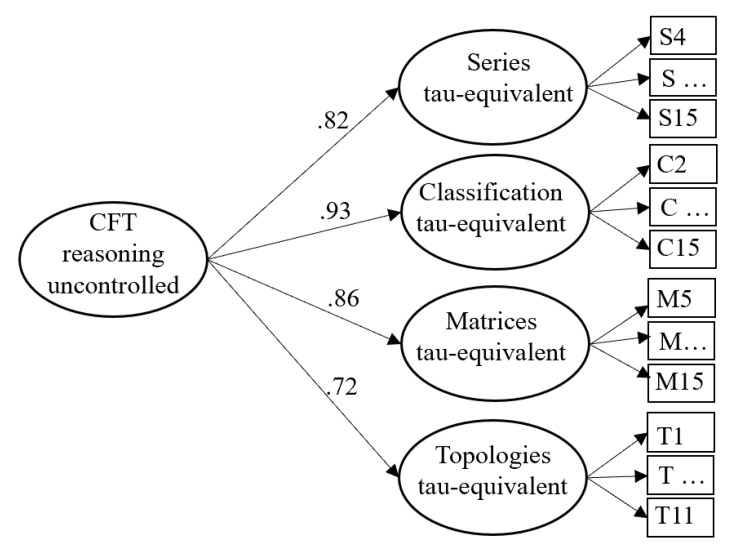
Measurement model of the four CFT subtests without controlling for the effect of speededness. For each subtest, one latent variable with constant factor loadings was extracted. From those four inductive reasoning latent variables, a second-order latent variable was extracted to represent inductive reasoning across the four CFT subtests.

To answer the first research question, we investigated the relationship between mental speed and reasoning ability uncontrolled for the effect of speededness. Four latent variables were extracted from the items of the four subtests of the CFT by means of tau-equivalent models, which were aggregated in terms of a second-order latent variable to represent reasoning ability uncontrolled for the effect of speededness (see Figure 1). The first three items of the Series subtest, the first item of Classification, and the first four items of Matrices were excluded from the analyses because they were solved by at least 98.5% of the participants; thus, their variances were too restricted. This model described the CFT data well, χ^2^(1120) = 1584.96, RMSEA = .046, SRMR = .084, AIC = 7161.42.

**Figure 2:**
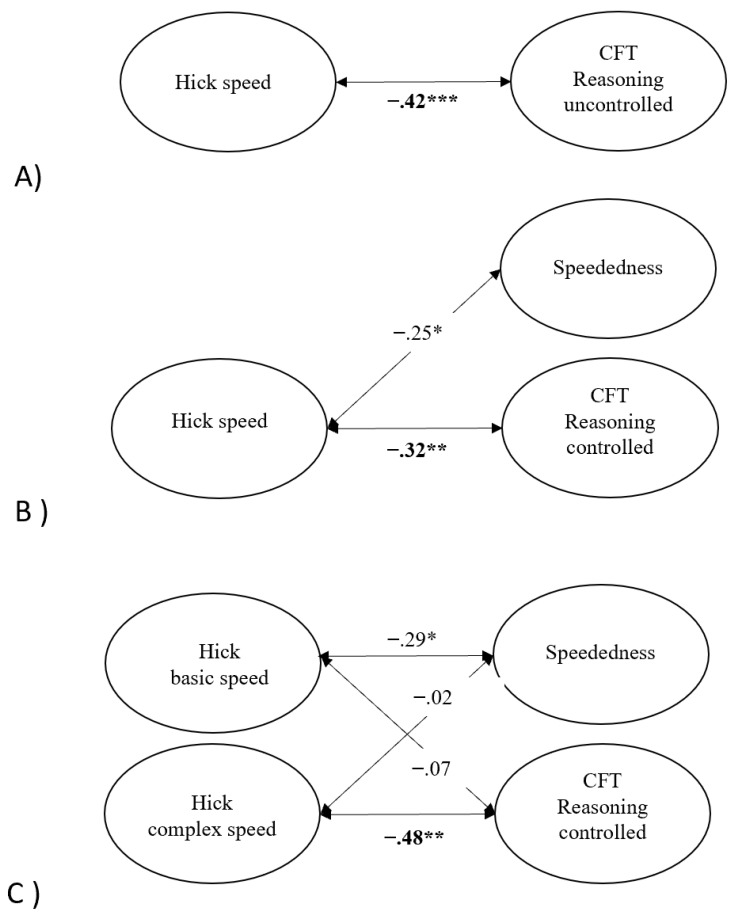
Latent structural equation models assessing the relationship (1) between mental speed and reasoning ability uncontrolled for complexity and speededness (**A**), (2) between mental speed, reasoning ability controlled for speededness, and speededness (**B**), and (3) between basic and complexity-related mental speed, reasoning ability controlled for speededness, and speededness (**C**). * *p* < .05, ** *p* < .01, *** *p* < .001.

Additionally, a latent variable from RTs in the three conditions of the Hick task was extracted to represent mental speed. The fit of this model is not reported as one latent variable extracted from three conditions of a task has no degrees of freedom. The latent variable was added to the measurement model of the CFT uncontrolled for the effect of speededness. In this model (Model 1), reasoning ability correlated negatively and significantly with the latent variable derived from RTs in the Hick task, *r* = −.42, *p* = .001, as presented in Panel A of Figure 2. The model showed an acceptable model fit, χ^2^(1263) = 1776.50, RMSEA = .045, SRMR = .083, AIC = 7648.61.

**Figure 3:**
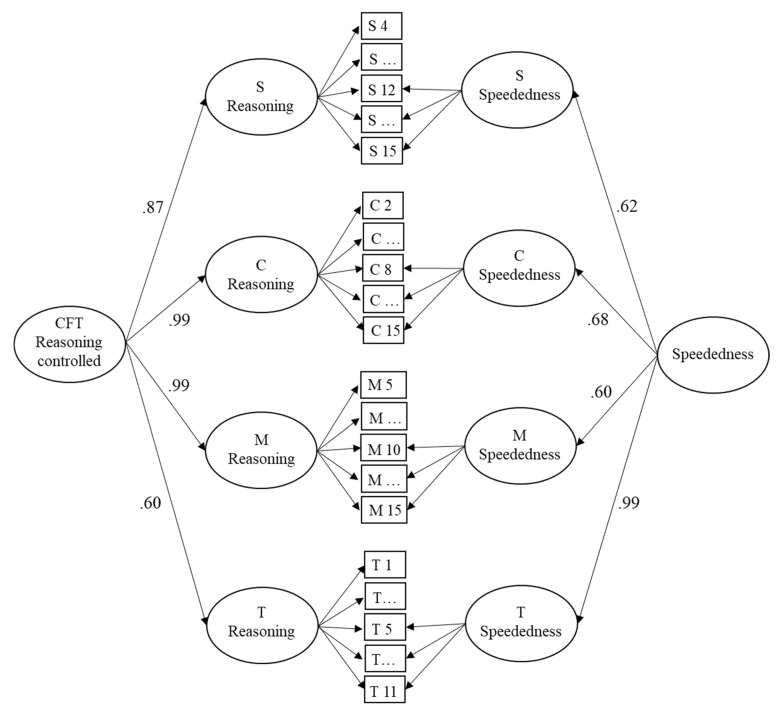
Measurement model of the four CFT 20-R subtests. For each subtest, one latent variable with constant factor loadings (and a fading-out effect at the end) represents reasoning and one latent variable with logistically increasing factor loadings represents the effect of speededness. From both the four inductive reasoning and the four speededness latent variables, second-order latent variables were extracted to represent inductive reasoning and the effect of speededness across the four CFT 20-R subtests. Note: S = Series; C = Classifications; M = Matrices; T = Topologies.

To examine how the correlation between reasoning ability and mental speed changes when the reasoning measure is controlled for the effect of speededness, a second measurement model was built as an alternative description of the CFT data. This model considered the effect of speededness in the four CFT subtests. Thus, from the responses on each of the four subtests, two latent variables were extracted by means of bifactor models. For the first latent variable, the factor loadings were fixed to 1 while, for the second latent variable, factor loadings were fixed in an increasing manner from the first item affected by speededness to the last item (see Section 2.5). The four first-order latent variables representing subtest-specific reasoning ability were combined to a second-order latent variable (overall reasoning ability). Analogously, the four first-order latent variables representing the effect of speededness in the four subtests were combined to create a second-order latent variable (overall speededness effect). This second measurement model is presented in Figure 3. It described the data well, χ^2^(1112) = 1462.47, RMSEA = .04, SRMR = .077, AIC = 7054.94, and better than the first measurement model of the CFT as can be seen from the lower AIC. It should be noted that the variances of the second-order latent variables were significant on the 1% level.

As for Model 1 in the previous section, we added the latent variable of mental speed extracted from the RTs in the Hick task to the second CFT measurement model. The resulting model (Model 2) showed an acceptable model fit, χ^2^(1254) = 1654.31, RMSEA = .040, SRMR = .076, AIC = 7544.43. As illustrated in Panel B of Figure 2, reasoning ability still correlated significantly with mental speed in the Hick task, *r* = −.32, *p* < .01, and the magnitude of the association was similar to that of the model without the speededness factor (*r* = −.42). In addition, the correlation between mental speed in the Hick task and the overall effect of speededness in Model 2 was statistically significant, *r* = −.25, *p* < .05.

### 3.2. The Effect of Increasing Task Complexity on the Relationship between Mental Speed and Reasoning Ability Controlled for the Effect of Speededness

The second research question was about the effect of increasing complexity of the ECT on the relationship between mental speed and reasoning ability when the measure of reasoning ability was controlled for the effect of speededness. To answer this question, we isolated complexity-related aspects of mental speed in RTs of the Hick task from basic aspects of mental speed by means of the above-described fixed-links model. This measurement model of RTs fit the data well, χ^2^(1) = 0.793, RMSEA = .001, SRMR = .026, AIC = 506.64. The latent variance of both latent variables was significantly different from zero (Hick basic speed *ϕ* = 0.105, *p* < .001; Hick complex speed *ϕ* = 0.012, *p* < .001).

The measurement model of the Hick task was combined with the measurement model of the CFT data, where the effect of speededness was controlled for. The resulting structural equation model yielded an adequate fit, χ^2^(1253) = 1649.88, RMSEA = .04, SRMR = .076, AIC = 7541.99. The structural core of this model is shown in Panel C of Figure 2. Unstandardized and standardized factor loadings are available in the supplementary material. Reasoning ability controlled for the effect of speededness was not related to basic aspects of mental speed, *r* = −.07, *p* = .59, but to the complexity-related aspects of mental speed in the Hick task, *r* = −.48, *p* < .01. The effect of speededness, on the contrary, was related to basic aspects of mental speed, *r* = −.29, *p* <.05, but not to its complexity-related aspects, *r* = −.02, *p* = .89, (see Figure 2C).

## 4. Discussion

The aims of the present study were, first, to investigate the influence of the effect of speededness in a reasoning test (CFT) on the relationship between reasoning ability and mental speed and, second, to evaluate the relevance of ECT (Hick task) complexity in this context. Our expectations were twofold: First, we expected that the association between reasoning ability and mental speed would become weaker when speededness due to the time limitation in the CFT was controlled for. Second, we expected that, when speededness in the CFT was controlled for, the association between reasoning ability and mental speed would be higher for complexity-related aspects of mental speed than for basic mental speed.

Replicating previous studies, CFT scores and RTs in the Hick task were substantially correlated when ignoring the effect of speededness in the CFT and the different levels of complexity in the Hick task (*r* = −.42). However, when speededness caused by the time limitation in the CFT subtests was statistically separated from reasoning ability using bifactor modeling (Ren et al. 2013, 2018; Zeller et al. 2018), the relationship between reasoning ability and mental speed decreased only slightly (to *r* = −.32), with the effects in both models being of medium size. These results speak against our first hypothesis but are in line with Vernon and Kantor (1986) who reported that the correlational relationship between intelligence and mental speed did not substantially change depending on whether intelligence was assessed under time-limited or time-unlimited conditions. Our result differs from Wilhelm and Schulze (2002) who compared the correlation between mental speed and reasoning ability in conditions of a time-unlimited and a time-limited administration of reasoning tests. In their study, the relationship between reasoning ability and mental speed was significantly weaker when the assessment of reasoning ability was time-unlimited compared to the time-limited conditions. However, in both conditions of Wilhelm and Schulze’s (2002) study, the correlation between reasoning ability and mental speed was significant, which corresponds to our finding that reasoning ability and mental speed are related even when speededness is controlled for. Thus, the general association between mental speed and reasoning ability (or intelligence) reported in the literature cannot be attributed to the effects of speededness alone. However, as the effect of speededness in the present study was related to mental speed (in almost the same strength as reasoning ability), failing to control for it might easily lead to an overestimation of the relationship between mental speed and reasoning ability or intelligence as also emphasized by Wilhelm and Schulze (2002).

Regarding the second hypothesis, reasoning ability (controlled for speededness) was unrelated to basic aspects of mental speed (*r* = −.07), but showed a robust correlation with complexity-related mental speed (*r* = −.48). Our hypothesis was, thus, supported. The separation of complexity-related from basic aspects of speed of information processing sheds further light on the question of how mental speed and reasoning ability are related when the effect of speededness is controlled for. Specifically, reasoning ability (controlled for speededness) appears to be associated only to those mental speed aspects that are required for handling the increasing demands on decision making in the Hick task. This result resembles Vernon and Kantor’s (1986) finding that time-unlimited intelligence tests correlated most strongly with RTs in the most complex ECTs (as defined on the basis of mean RTs). The effect of speededness, however, was only related to basic and not to complexity-related aspects of mental speed in the present study. These basic aspects of mental speed probably reflect processes of sensory encoding and motor execution as well as individual differences in alertness, fatigue, or motivation (Heitz et al. 2005; Rammsayer et al. 2017) that impact performance in both the Hick task and the CFT.

Thus, the answer to the question of whether a time limitation in the administration of an intelligence test impacts the relationship between intelligence and mental speed probably depends on the complexity of the ECT to measure mental speed. In the case of a very simple, undemanding ECT, a time limitation in the reasoning test probably leads to an overestimation of the mental speed–reasoning ability relationship. RTs from more complex and cognitively demanding ECTs, on the contrary, correlate with reasoning ability even more strongly when no time limitation is used during the administration of the reasoning test or when the effect of speededness is controlled for.

A potential explanation for this differential relationship between basic and complexity-related aspects of mental speed, on the one hand, and reasoning ability and the effect of speededness, on the other hand, has been proposed by Wilhelm and Schulze (2002). According to their “two-functions view”, mental speed tasks and reasoning tasks can be organized along a complexity dimension with mental speed (and low working memory demands) at the one pole and reasoning (with high working memory and low mental speed demands) at the other pole (Wilhelm and Schulze 2002). The correlation between two cognitive measures is determined by how close they are to each other on the complexity dimension. Reasoning ability controlled for speededness and basic mental speed controlled for complex mental speed are not expected to correlate as they are at the extreme poles of the complexity dimension. This is what we found in the present study; basic aspects of mental speed in the Hick task were not related to reasoning ability controlled for speededness. According to the “two-functions view”, however, a correlation between mental speed and reasoning is expected in two cases: first, when speededness is introduced into the reasoning measure, because in this case, reasoning also contains aspects of basic speed, and second, when the task demands in the ECT are increased and RTs not only represent basic but more complex mental speed. Both these assumptions of Wilhelm and Schulze’s (2002) two-functions view were empirically confirmed in the present study. First, the effect of speededness was related to basic mental speed extracted from the Hick task and, second, complexity-related aspects of mental speed extracted from the Hick task were related to reasoning ability controlled for the effect of speededness.

Taken together, the well-established relationship between mental speed and reasoning ability might be influenced by time limitations of tests assessing reasoning ability. The present study demonstrates that this influence can be controlled for by means of bifactor CFA models. Mental speed is related to both reasoning ability and the effect of speededness. However, the effect of speededness in the reasoning measure is related to basic aspects of mental speed while reasoning is substantially related to complexity-related aspects of mental speed.

Future studies are needed to examine whether time limitations and resulting speededness effects also affect the magnitude of the association between mental speed and other tests of reasoning ability as well as broader assessments of psychometric intelligence (e.g., Wilhelm and Schulze 2002). Our findings may also be relevant when choosing and interpreting reasoning or intelligence tests in applied contexts. Specifically, the predictive validity of reasoning scores for outcomes such as academic performance might change when the effects of speededness are controlled for. Implications of the present research for everyday-life correlates of intelligence, however, need to be examined explicitly.

It should be noted that the latent variable representing the effect of speededness is probably not a pure measure of this effect. Item processing does not only change from the earlier to the later items due to time limitations but also due to other processes. For example, individual differences in learning the rules underlying the items develop from item to item (Ren et al. 2014; Schweizer et al. 2021) and effects of increasing fatigue are also plausible (Lozano and Revuelta 2020). Individual differences in managing proactive interference (May et al. 1999) have been described to influence performance on Raven’s Advanced Progressive Matrices (Bunting 2006) and can be expected to increase from earlier to later items. A systematic change during test completion has also been reported for processing strategies (Gonthier and Roulin 2020). All of these processes might have contributed to the latent variable, which we call the effect of speededness. The logistic function underlying the course of factor loadings was used to depict this effect as purely as possible, but it seems unlikely that this procedure completely excludes other processes from this latent variable. However, regardless of the purity of the latent variable representing the effect of speededness, we assume that the influence of speededness on the latent reasoning variable could be successfully controlled for.

Regarding the decomposition of individual differences in mental speed measures, it would be interesting to compare the fixed-links modeling approach of the present study with other contemporary methods, such as the diffusion model (Schmitz and Wilhelm 2016), ex-Gaussian distribution (Schmitz and Wilhelm 2016), or the linear ballistic accumulator model (Brown and Heathcote 2008).

Certainly, the present findings should be replicated and extended by using other ECTs or Hick task conditions with higher complexity than that in the present study, in which the most complex condition (2-bit Hick task) was still quite simple. Finally, our findings are based on a sample with a relatively high mean level of reasoning (see above). It would be interesting to replicate and generalize the present findings in samples with a broader range of intellectual abilities.

## Figures and Tables

**Table 1:** Mean (*M*), Standard Deviation (*SD*), Minimum (*Min*), Maximum (*Max*), Skewness, and Kurtosis of the number of correct responses in the four CFT subtests as well as of the reaction times in the three conditions of the Hick task and the Flanker task.

	*M*	*SD*	*Min*	*Max*	Skewness	Kurtosis
CFT						
Series	9.54	1.84	4	12	−0.58	−0.21
Classification	10.27	2.02	5	14	−0.35	−0.69
Matrices	7.66	1.73	3	11	−0.10	−0.43
Topologies	7.26	1.77	1	11	−0.60	0.56
Reaction times in the Hick task [ms]						
0-bit condition	263	41	208	420	1.92	4.69
1-bit condition	321	45	238	450	1.31	2.71
2-bit condition	402	67	224	600	0.87	0.96

## Data Availability

The data presented in this study are available in Supplementary Material.

## Supplementary Materials

The following supporting information can be downloaded at: https://www.mdpi.com/article/10.3390/jintelligence11050089/s1, Table S1: Unstandardized and standardized factor loadings of the model with basic and complexity-related mental speed, reasoning ability controlled for speededness, and speededness. Data, Script.
