# Supplementary material for: How Speededness of a Reasoning Test and the Complexity of Mental Speed Tasks Influence the Relation between Mental Speed and Reasoning Ability
Source: J Intell. 2023 May 8;11(5):89. doi: 10.3390/jintelligence11050089 (PMC10219451; doi:10.3390/jintelligence11050089)
Supplement: Supplementary file 1 [file jintelligence-11-00089-s001.zip › readme.pdf]

This document provides the codebook for all variables in the dataset used in the study titled "How speededness of a reasoning test and the complexity of mental speed tasks influence the relation between mental speed and reasoning ability".

Vpn	anonymous identifier of participant
age	age in years
Intelligenz	IQ based on CFT
CFT	sum score CFT
Sex	1 = female; 2 = male
H0MW	mean response latency of first Hick task [in milliseconds]
H1MW	mean response latency of second Hick task [in milliseconds]
H2MW	mean response latency of third Hick task [in milliseconds]
A1-A15	Items of Series (CFT) 0 = incorrect; 1 = correct
B1-B15	Items of Classifications (CFT) 0 = incorrect; 1 = correct
C1-C15	Items of Matrices (CFT) 0 = incorrect; 1 = correct
D1-D11	Items of Topologies (CFT) 0 = incorrect; 1 = correct
