# Supplementary material for: How Speededness of a Reasoning Test and the Complexity of Mental Speed Tasks Influence the Relation between Mental Speed and Reasoning Ability
Source: J Intell. 2023 May 8;11(5):89. doi: 10.3390/jintelligence11050089 (PMC10219451; doi:10.3390/jintelligence11050089)
Supplement: Supplementary file 1 [file jintelligence-11-00089-s001.zip › Table S1. Unstandardized and standardized factor loadings.pdf]

Supplementary Materials: How Speededness of a Reasoning Test and the Complexity of Mental Speed Tasks Influence the Relation between Mental Speed and Reasoning Ability

Table S1. Unstandardized and standardized factor loadings of the model with basic and complexity-related mental speed, reasoning ability controlled for speededness, and speededness.

	**S Reasoning**		**S Speededness**	
	**unstandardized**	**standardized**	**unstandardized**	**standardized**
S4	0.331	0.252		
S5	0.184	0.255		
S6	0.207	0.259		
S7	0.279	0.254		
S8	0.418	0.258		
S9	0.392	0.261		
S10	0.444	0.258		
S11	0.357	0.255		
S12	0.400	0.263	0.151	0.207
S13	0.460	0.256	0.287	0.332
S14	0.477	0.264	0.390	0.451
S15	0.500	0.261	0.462	0.503
	**C Reasoning**		**C Speededness**	
	**unstandardized**	**standardized**	**unstandardized**	**standardized**
C2	0.184	0.196		
C3	0.140	0.193		
C4	0.196	0.188		
C5	0.342	0.192		
C6	0.347	0.196		
C7	0.352	0.189		
C8	0.436	0.197	0.165	0.121
C9	0.466	0.195	0.290	0.198
C10	0.483	0.197	0.395	0.263
C11	0.436	0.192	0.403	0.289
C12	0.462	0.200	0.449	0.317
C13	0.444	0.201	0.439	0.325
C14	0.497	0.200	0.495	0.325
C15	0.325	0.182	0.324	0.296
	**M Reasoning**		**M Speededness**	
	**unstandardized**	**standardized**	**unstandardized**	**standardized**
M5	0.424	0.157		
M6	0.313	0.155		
M7	0.263	0.155		
M8	0.342	0.158		
M9	0.407	0.157		
M10	0.430	0.156	0.162	0.197
M11	0.380	0.157	0.237	0.329
M12	0.492	0.157	0.402	0.432
M13	0.472	0.159	0.436	0.495

M14	0.496	0.157	0.481	0.512
M15	0.352	0.154	0.348	0.512
	**T Reasoning unstandardized**	**standardized**	**T Speededness unstandardized**	**standardized**
T1	0.156	0.280		
T2	0.384	0.273		
T3	0.449	0.280		
T4	0.184	0.277		
T5	0.331	0.279	0.125	0.128
T6	0.319	0.288	0.199	0.218
T7	0.466	0.281	0.381	0.279
T8	0.474	0.283	0.438	0.318
T9	0.492	0.276	0.477	0.326
T10	0.367	0.271	0.363	0.326
T11	0.307	0.272	0.305	0.330
	**Hick basic speed unstandardized**	**standardized**	**Hick complex speed unstandardized**	**standardized**
Hick 0-bit	1	0.808	1	0.280
Hick 1-bit	1	0.758	2	0.525
Hick 2-bit	1	0.495	4	0.685
